# Application of Ecological Momentary Assessment in Studies with Rotation Workers in the Resources and Related Construction Sectors: A Systematic Review

**DOI:** 10.1016/j.shaw.2022.10.004

**Published:** 2022-10-15

**Authors:** Bernard Yeboah-Asiamah Asare, Suzanne Robinson, Dominika Kwasnicka, Daniel Powell

**Affiliations:** 1 Curtin School of Population Health, Curtin University, Bentley, Australia; 2 Health Psychology, Institute of Applied Health Sciences, University of Aberdeen, Aberdeen, United Kingdom; 3 Deakin Health Economics, Institute for Health Transformation, Deakin University, Burwood, Australia; 4 Faculty of Psychology, SWPS University of Social Sciences and Humanities, Wroclaw, Poland; 5 NHMRC CRE in Digital Technology to Transform Chronic Disease Outcomes, Melbourne School of Population and Global Health, University of Melbourne, Melbourne, Australia; 6 Rowett Institute, University of Aberdeen, Aberdeen, United Kingdom

**Keywords:** Ecological momentary assessment, FIFO, Mining, Offshore oil and gas, Rotation work

## Abstract

Whilst Ecological momentary assessment (EMA) can provide important insights over time and across contexts among rotation workers whose work periods alternate with leave at home, it can also be challenging to implement in the resources and construction sectors. This review aimed to provide a summary of the methodological characteristics of EMA studies assessing health outcomes and related behaviors in rotation workers. Systematic searches in PubMed, Medline, EMBASE, CINAHL, PsycINFO, and Scopus were done to include 23 studies using EMA methods in assessing health-related outcomes and behaviors. EMA designs included daily diary: assessments once per day typically fixed at the end of day (47.8%), within day fixed interval time-based design: assessments on multiple times per day at certain times of day (17.4%) and combination of both designs (34.8%). Studies employed paper and pencil diaries (73.9%) and one or more electronic methods (60.9%): wrist-worn actigraphy device (52.2%) and online-based diaries (26.1%) for data collection. Most of the studies (91.3%) did not report prompting -EMAs by schedule alerts or compliance. Daily diary and within day fixed interval dairies designs are common, with the increasing use of electronic EMA delivery techniques. It is unclear how well participants adhere to assessment schedules, as these are inadequately reported. Researchers should report compliance-related information.

## 1 Introduction

Rotation work arrangements have become the standard model in the resources sector [1]. Rotation work, also known commonly as *Fly-In Fly-Out* (FIFO), involves workers traveling to remote areas, being accommodated and provided with food to work for a specified number of days and to return home to spend another fixed number of days [1].

Rotation work has some benefits both for the companies (e.g., by reducing the cost of establishing and operating mine sites) and workers (e.g., higher wages and extensive leave periods to spend time with family and friends) [1]. However, there are some concerns that rotation work may be associated with poorer health outcomes, such as higher psychological distress [2], sleep and fatigue problems [3], and health-related behavioral problems such as higher alcohol consumption and smoking [4].

Rotation workers lifestyles are characterized by distinct contexts and routines during on-shift and off-shift days [5]. For instance, during on-shift days, rotation workers work long hours, live and work in remote areas far away from their families and social networks, usually without their domestic or family obligations [5]. On off-shift days, workers are free from work commitments but take up their family roles [5]. Requirements of this distinct lifestyle make workers take up ‘different social roles and patterns of behavior (p3) [5]. Studies that employ methods that examine within-person processes across time and everyday life contexts of rotation workers are needed. Ecological momentary assessment (EMA) studies can demonstrate important insight into the daily life of rotation workers, how they experience various health variables day-to-day, and what features of their working lives predict such variability. EMA provides the opportunity to examine how the outcomes and predictors vary and covary within-persons, over time, and across contexts, as people go about their usual daily activities [6,7] and has the potential to assess the health outcomes and experiences of rotation workers with precision. The advantages of EMA methods over the traditional research methods include reduced self-report bias as recent/current states are assessed, greater ecological validity as assessments is done in subjects' natural environment, and the repeated assessments assist in understanding the variations of experiences and behavior over time and across settings [7]. With the advancement in mobile technologies, EMA has become a flexible research design with various options for the scheduling of assessments, including assessments that are event-contingent (e.g., individuals reporting as and when they experience an event such as pain), time-contingent (fixed, random, or quasi-random) (e.g., individuals signaled at particular time intervals in the day to report on the number of standard drinks consumed) [7,8], or in the case of wearable sensors (e.g., accelerometry), assessments may be continuous and reliant only on wear time.

As technological capabilities increase to observe behaviors and other phenomena in daily life, opportunities to carry out EMA studies with rotation workers are also likely to increase. Available EMA studies provide a useful insight into rotation workers lives (e.g., [6,[9], [10], [11], [12]]), as the suitability of the EMA methodology allows us to monitor the workers as they are on and off shifts in their ‘natural work settings’. Despite the advantages of EMA methods, there are some challenges, including the burden on participants [13], participants' compliance to study protocol and missing data [14], low sample sizes [15], and can be expensive [15], all of which may make it difficult to implement in some populations. As such, considerations about the suitability of different EMA study design choices become necessary [7] to make informed decisions.

In many ways, rotation work in the resources sector is unique and may present challenges for the application of EMA methods, such as multiple and randomized time-based assessments. Rotation workers typically have a routine day of daily alcohol and random drug testing, working compressed day and night shifts of a standard of continuous 12 hours starting and ending at pre-specified times, with short snacks and fatigue breaks: a work schedule that interfere with regular behaviors/activities including eating, sleeping, and social interactions [16]. Rotation workers also may operate heavy machinery and work in noisy and critical safety environments, which require specified or prescribed and standardized safety apparel (personal protective equipment) all the time, with full attention on tasks and potentially limited access to personal or other mobile devices with internet connections to which EMA studies may be deployed [17].

There is therefore the need to understand how to best utilize this method to learn about this population, design comprehensive studies that allow us to make conclusions about health, and design just-in-time adaptive interventions that support workers health when they are at work and at home. EMA approaches to assessing the health outcomes in rotation workers have not been comprehensively assessed. Considering that the use of EMA is comparatively a new approach for examining the health outcomes among shift and rotation workers, concerted efforts are required to improve the key aspects of EMA studies and methods so that their use could be consistent and replicated in rotation work and other settings. In this review, we aim to provide a summary of the methodological characteristics of EMA studies [e.g., EMA design/strategy and assessment schedules (design, monitoring period, study duration), sampling and measures, EMA delivery method—technology and administration (data collection methods), response and protocol compliance, and data analysis plan] assessing health outcomes and related health -behaviors in resource and construction industry rotation workers. These methodological characteristics are critical considerations in the implementation of EMA studies and the documentation of such features will permit better evaluation of EMA studies and their findings, and the appropriateness of the different methods/procedures applied in assessing particular phenomena and study populations [18]. For instance, a previous review found the use of paper-and-pencil and fixed-schedule designs as most common in assessing the psychological and behavioral experiences in older adult populations [19]. Another systematic review also found differences in EMA designs employed in assessing diet and physical activity among the youth population, with studies employing both paper-and-pencil and electronic EMA designs and mostly interval-contingent prompting strategies [20]. Furthermore, a previous review assessing the compliance to mobile-EMA protocols has suggested that the EMA study design use may affect compliance in the youth population in different settings [21]. It is also suggested that the findings of EMA studies could be misinterpreted if the key aspects of the EMA design employed and participant compliance are not provided, and as such recommended for more consistent EMA reporting [20]. The learnings from this review about the implementation of EMA in rotation workers in the resource and construction industry will guide future EMA studies and are potentially transferable to other worker populations engaging in shift and rotation work.

## 2 Method

### 2.1 Eligibility criteria

Studies were included if (1) original articles were published in peer-reviewed journals and English; (2) participants were rotation workers and worked in the resource (offshore oil and gas, and mining) and construction industry; (3) studies employed EMA designs, including event- or signal-time-based sampling, continuous assessment, and daily surveys; (4) used EMA-based techniques including any electronic, wearable, or mobile technology (such as cell/smart-phones, handheld devices, PDAs); website/online diaries/surveys and paper-based diaries/surveys for data collection; (5) assessed mental health and physical health outcomes, sleep problems (sleep duration and quality, sleepiness and fatigue), or health-related behaviors including alcohol intake, smoking and drug use, diet and physical activity, measured via EMAs and continuous assessments.

Studies were excluded if (1) they were reviews, letters, editorials; (2) EMA designs and strategies were not clearly defined; (3) there were no repeated measures and/or variable collected momentary or diary data once in less intensive frequency than weekly intervals during the study period; (4) data collection was done in a laboratory setting or not in participants' real-life natural living environment; (5) they reported on adaption and re-adaption of circadian rhythm to shift patterns measured by cortisol concentration and *6-sulphatoxymelatonin acrophase*.

### 2.2 Literature sources and search strategy

Literature searches were conducted in the databases: PubMed, Medline, EMBASE, CINAHL with Full Text, PsycINFO, PsycArticles, and Scopus for relevant articles on 1st May 2020 as part of a bigger review [3] assessing rotation workers health in the resource industry [pre-registered on PROSPERO (ID=CRD420-20167649)] and updated on 21st April 2021. The search strategy comprised terms linked to health and FIFO work, with no restrictions on study design, publication dates, and geographic location but restricted to peer-reviewed articles and those published in English language set [3]. An additional hand search of the references of the included studies was also done for other relevant studies.

### 2.3 Study screening and selection process

To reviewers, BYAA and DK independently screened the titles/abstracts and later full text of articles for eligibility and inclusion into the review, and any inconsistencies in the selection were discussed and resolved by consensus. The systematic review of the literature was reported in line with the Preferred Reporting Items for Systematic Reviews and Meta-Analyses guidelines [22]; the detailed study selection process is presented in Fig. 1 (Supplementary Material 1).

### 2.4 Data extraction and data items

Data were extracted using a data extraction sheet developed according to the Checklist for Reporting EMA Studies [20] and other reporting guidelines [18]. The key information extracted included study authors, publication year, study design, aims/objectives, study setting (country and industry) and participants (sample size, age), and health outcomes. We also extracted the main EMA methodological characteristics including EMA design/prompting strategies (event-or signal-based contingent), method/technology used for data collection, monitoring period (number of data collection wave), study duration, prompt frequency, protocol compliance, measures used to assess outcome understudy (number of items used and validity), and data analysis (model type). Data extraction was done by one reviewer (BYAA) and another (DK) double-checked 10% of the data, and the cases of inconsistencies were resolved through the discussions.

### 2.5 Data synthesis

Data extracted were descriptive and were presented in tables based on study characteristics (author/year, setting/country, sample size, analytical sample, age, study type, outcomes, and predictors) and EMA methods features (EMA design/approach, method for EMAs delivery, monitoring periods/Study duration, compliance rate/compliance enhancer, assessment frequency, assessment period, outcomes measures, and analysis method). Data were narrative synthesized under the following areas: characteristics of studies, sampling and measures, EMA design/strategy and assessment schedules, EMA delivery method-technology and administration, response and compliance, and analytical methods.

## 3 Results

### 3.1 Study selection

The searches retrieved 6978 records, and after removing duplicates, 86 studies were screened at full text for eligibility. Twenty-three (23) studies were included in the review (Fig. 1).

### 3.2 Characteristics of studies

The included studies were published between 1998 and 2021 and conducted in the United Kingdom (*n* = 6; 26.1%), Australia (*n* = 6; 26.1%), Norway (*n* = 5; 21.7%), the Netherlands (*n* = 4; 17.4%), Thailand (*n* = 1; 4.3%), and Iran (*n* = 1; 4.3%). The majority of studies (*n* = 17) recruited participants from the oil and gas workers, 4 from mining workers and 1 each with FIFO workers (predominately mining workers) and construction rotation workers. The characteristics of the included studies are summarized in Table 1 (Supplementary Material 2).

### 3.2 Sampling and measures

The average number of participants recruited per study was 54.0 ± 31.7 (range 7–111) and included an average analytical sample of 34.7 ± 17.6 (range 6–64). The mean age of study participants was 41.2 ± 3.0 years (range 35.9–47.5). One of the studies did not report the mean age of participants [23].

Of the 23 studies, 19 (82.6%) studies assessed sleep and fatigue [6,[9], [10], [11], [12],[24], [25], [26], [27], [28], [29], [30], [31], [32], [33], [34], [35], [36], [37]], 3 (13%) studies assessed mental health outcomes including emotional exhaustion and engagement [21], depression and anxiety [37]; 5 (21.7%) studies assessed health-related behaviors including exercise/physical activity and relaxation [6,11,12,38], alcohol intake [6,11,39], smoking [6,39] and eating behavior [6]; 1 (4.3%) study measured physical health status [6]. All included studies used self-reported measures to assess outcomes; 14 studies [9,10,12,[25], [26], [27], [28], [29], [30], [31], [32], [33],^35^,^36^] combined self-reported EMAs with additional objective measurements. The number of self-reported items used was dependent on the outcome being measured; for instance, sleepiness or fatigue were mostly assessed using a single item whereas sleep outcomes were mostly assessed using questionnaires with multiple items.

### 3.3 EMA design/strategy and assessment schedules

All included studies used a regular time-based approach: daily diary design *(assessment once per day typically at end of day)* (*n* = 11; 47.8%) [6,[23], [24], [25],[28], [29], [30], [31],[37], [38], [39]], within day fixed interval diaries design *(assessments on multiple times per day at certain times of day)* (*n* = 4; 17.4%) [9,12,26,27], and 8(34.8%) studies combined daily diary design with within day fixed interval diaries design [10,11,[32], [33], [34], [35], [36],^40^] (Table 2) (Supplementary Material 2).

All of the included studies assessed an outcome at regular interval schedules; 19 (82.6%) studies assessed an outcome once per day (morning or evening) [6,11,[23], [24], [25],[28], [29], [30], [31], [32],[34], [35], [36], [37], [38],^40^], 8 (34.8%) of the studies [9,11,26,27,33,36] collected data twice daily (before and after work shifts or morning and evening), 3 (13%) studies [23,33,39] collected data once or twice daily in every 3 days, 4 (17.4%) studies carried out hourly assessments for one of the main study outcomes measure (sleepiness) [32,34,35,40], and 12 (52.2%) studies also combined regular interval dairy assessments with continuous monitoring for assessing sleep outcomes and physical activity [9,12,[26], [27], [28], [29], [30], [31],^35^,^36^].

The majority of studies (*n* = 21; 91.3%) did not report whether or not the participants were in any way prompted (reminded) to complete assessments. Only two studies reported prompting participants using alerts via smartphones; one study sent text messages once daily to remind participants to complete their diary assessments [23] and another sent prompts but did not report their frequency [32].

The majority of studies (*n* = 18; 78.3%) monitored study participants and collected data over one monitoring period (i.e., one wave of data collection) [9,11,12,23,26,28,30,31,35,36,[38], [39], [40], [41]]. Four studies [6,29,32,34] collected data over 2 monitoring periods whereas one study [37] had 6 data collection periods/waves. Data were collected for an average of 18.1 ± 7.5 consecutive days (range 7–28) per single wave of data collection: 7 days (3 studies), 11 days (1 study), 14 days (8 studies), 21 days (2 studies), 22 days (2 study), and 28 days (6 studies). Studies with burst designs with more than one monitoring period collected data for an average of 50.4 ± 25.4 total days (range 14–84 days). Data collection was undertaken during both on- and off-shift rotation periods in 11 studies (47.8%), on-shift only in 9 studies (39.1%) and off-shift only in 3 studies (13%).

### 3.4 EMA delivery method—technology and administration

The majority of studies (*n* = 17; 73.9%) used paper and pencil diaries and 14 (60.9%) studies used electronic methods: website/online-based diaries (*n* = 6; 26.1%) [6,9,12,23,26,27], wrist-worn device (actigraphy) (*n* = 12; 52.2%) [9,10,12,[25], [26], [27], [28], [29], [30], [31],^35^,^36^], and hand-held device (*n* = 4; 17.4%) [27,32,33,35]. Eight of the studies used both paper and pencil diary and electronic methods: wrist-worn device (actigraphy) [10,25,[28], [29], [30], [31]] for assessing sleep outcomes and hand-held computer for assessing sleepiness/fatigue [10,32,35]. Four of the studies combined two electronic methods: website/online-based diaries alongside a wrist-worn device (actigraphy) [9,12,26,27] for assessing daily sleep outcomes and *reaction time task* delivered through an iPad to assess cognitive fatigue [27]. Studies that used website/online-based diaries were delivered to participants through emails containing links to surveys [6,9,23,26,27].

### 3.5 Response and compliance

Compliance is defined as the percentage of scheduled assessments to which participants responded by completing the measurement [18]. Participation or response rate is defined as the percentage of participants who completed the predetermined number of assessments (i.e., all scheduled assessment days) [21]. Only two of the studies reported compliance rates; among these, compliance was 80.3% [38] and 87% [11] of total assessments. All the studies reported the sample size they had included in their analysis, and the response rate based on that, as a proportion of the recruited sample, ranged from 31.5 to 100.0% (mean = 70.3%). Three studies explicitly reported response rates of 67.8% [29], 95% [11], and a range of 66–78% across 6 waves of data collection [37]. Studies that reported a response rate of less than 50% were of either daily diary design [25] or combined both daily diary and interval diary designs [10,35], and collected data over 1 monitoring period and for an average of 18 days (range 14–22 days) [10,25,35]. Likewise, studies reporting a response rate of greater than 80% were either a daily diary design [6,31] or combined both daily diary and interval diary designs [11,33,34,40], and data collection was done over 1 monitoring period [11,31,33,40] or 2 waves (6,25) and for an average of 23.3 days (range 7–42 days) [6,11,31,33,34,40].

Sometimes, research using EMA may specify a compliance threshold (level of data completion) for inclusion in the study analysis. Most of the studies (*n* = 18; 78.3%) did not explicitly report compliance threshold [6,9,10,12,[24], [25], [26], [27], [28],[30], [31],[33], [34], [35],[37], [38], [39], [40]], while 5 studies reported level for data completion required for data inclusion: 100% compliance [36], at least 3 daily surveys [23], at least complete data for 1 week or more [29,32], and at least 1 day or night shift period [11]. Authors in most of the studies (*n* = 14; 60.9%) cited work arrangements, lost to follow-up, incomplete assessments, withdrawal, and personal reasons of participants such as sick/annual leaves and transfers for either non-compliance or dropout (participation) or exclusion from analysis.

Most of the studies (*n* = 19; 82.6%) did not report on any incentives or reimbursements to study participants in return for participation that might have been given; 3 studies explicitly reported not giving any incentives [34,35,40], and 1 (5%) study [6] reported giving personalized feedback on health outcomes to interested participants as an incentive. Few studies explicitly reported the use of any other compliance-enhancing strategy, with only daily remote monitoring for completion by investigators (*n* = 2; 10%) [26,32] and collection of paper diaries at the end of each day (*n* = 1; 4.3%) [32] stated as methods incorporated to encourage compliance.

### 3.6 Analytical methods

Most of the studies (*n* = 13; 56.5%) aggregated data to the person-level to create a summary metric and analyzed using analysis of variance and/or correlation or standardized parametric regression [11,[28], [29], [30], [31], [32], [33], [34], [35], [36], [37],^39^,^40^]. Some studies (*n* = 8; 34.8%) used linear mixed models or generalized linear mixed models [6,9,10,12,23,[25], [26], [27]] and 2 (8.7%) studies used generalized estimating equations [24,38].

## 4 Discussion

The main aim of this study was to systematically review and summarize EMA studies assessing several health outcomes and related behaviors in rotation workers to describe the common EMA methodological characteristics and discuss other methods that could be explored in this workgroup.

### 4.1 Sampling, EMA design/strategy, and assessment schedules

Included studies had varied and relatively low sample sizes, with an average of 54.0 ± 31.7 (range 7–111) participants recruited per study. The power to detect within-person effects is higher in EMA studies due to a large number of repetitive data points [15], which allows studies to typically recruit fewer participants [13]. The sample size is also a function of assessments, days of monitoring, etc., where studies with many assessments and longer monitoring periods may typically have low sample sizes. For instance, the current review studies that carried out hourly assessments per day and over an average of 33 days reported an average sample size of 20 (range 7–38). However, consequential of the burden on participants associated with EMA studies, fewer participants may also want to participate in the EMA studies, often resulting in low samples sizes compared to field surveys [15]. The burden of commitment required to complete repeated/several surveys [23,35] was stipulated to affect participation and attrition of rotation workers in the included EMA studies.

All studies included in this review employed regular-interval time-based designs; daily diaries and within-day fixed interval contingent diaries. Daily diaries which are a special type of time-based designs [7], involves assessments once per day typically fixed at the end of the day [13,42], whereas within-day fixed interval contingent designs involve assessments multiple times per day at certain, usually pre-specified, times of day [13,42]. The daily diary approach is easy to administer and less demanding on study participants [7,13], and within-day fixed interval signal-contingent designs are also considered less intrusive on study participants [13] than the other EMA designs; *random interval assessments*: involve multiple assessments per day at random schedules, and *event-based design*: involves assessments that are initiated by the occurrence of a predefined event of interest [7,8,13,42]. Daily diary and within-day fixed time-based designs seem more appropriate for rotation workers than variable time schedules of random assessments and event-based assessments [7]; as rotation workers in the resources sector work compressed day and night shifts of a standard of 12 hours and work schedule may not allow for multiple random assessments; workers could only be available to respond to assessments at fixed times which may coincide with their break times and/or after shift periods. Again, the daily diary design is deemed most appropriate for assessing outcomes that show no significant variation within the day [13,42], such as sleep. Evidence from our review showed most of the studies (≈83%) employing daily diary designs examined sleep outcomes. However, daily diary designs are subjected to recall bias as they rely on recalls to capture experiences over the day and may not be representative of the subject experiences [13,42]. Within-day fixed interval designs although lessen the biasing effect of end-of-day or bedtime assessments as in daily diaries due to the short recall periods [13], could also be susceptible to measurement reactivity where participants may alter their behaviors or experiences in anticipation of assessments [13].

Evidence in our review has suggested multiple assessments within a day among offshore rotation workers could be done during work periods (at every other hour) using single-item measures [32,34,35,40] and the involvement of the participant's organization [12]. These more-intensive study designs recorded response rates between 44.7 and 85.7%. Other designs choices such as *random interval assessments* and *event-based design* have not yet been used in rotation workers, and it is unclear whether this is due to being unsuitable for the population in general or the research questions selected.

Evidence from our review showed participants tended to be monitored over one wave. Possibly due to the demand of EMAs on study, participants [43] coupled with the demanding nature of rotation work arrangements. However, some studies demonstrated that data collection over 2 or more waves [29,32,35,37] could be applied among rotation workers and across the on-and off-shift phases [6,11,26,30,32] of the rotation work roster.

Studies in our review assessed most of the outcomes once per day but those assessing sleepiness/fatigue reported an assessment frequency of 2 or more times per day. Choosing the frequency of assessments in an EMA is guided by the level of variability of the phenomenon under study, the theoretical basis of the study and the burden on study participants [7,18]. A higher frequency of assessments per day affords better ‘temporal resolution’ of the phenomenon whereas assessment for several days may increase generalizability [18]. However, a higher assessment frequency could increase the invasiveness of the study [43] and burden on study participants [18]. Mechanisms suggested to reduce participants' burden include the use of electronic devices in EMA [44] and/or the brevity of items of measures [18]. Evidence from our review showed studies indicated using single and/or reduced items to reduce participants' burden [6,23]; studies with a higher frequency of assessments within a day employed single-item measures [32,34,35,40].

Evidence in our review suggests studies' prompting schemes were generally inadequately reported. Prompting schemes are usually used in EMA studies using time-based assessment schedules to alert study participants when assessments are to be completed [7], and evidence suggests prompting participants enhances compliance even with a paper diary protocol [45]. Future EMA studies among rotation workers should report on the prompting schemes used to guide the design of subsequent studies in rotation workers.

### 4.2 EMA delivery method—technology and administration

The use of paper and pencil diaries to deploy assessments were the most common in our review. Paper and pencil dairies may be easy to implement [46]. But due to the lack of time-stamped entries [46], paper and pencil diaries are limited by ‘hoarding’ (*failure to complete assessments at the specified time but later backfills the missed data*) [13,18], and high falsified compliance to scheduled assessments (*difference between participant's reported compliance and their objectively measured actual compliance to scheduled assessments*) [18,45]. Recent studies included in the review suggested the increasing use of electronic diaries [6,9,12,23,26,27]. Evidence in our review suggested study participants' preference for online diaries over paper and pencil diaries in onshore rotation workers [23]. Compliance with using paper and pencil diaries and electronic diaries in our review were inadequately reported. However, electronic diaries have been demonstrated to produce higher participants' compliance [47] than paper and pencil diaries. The use of mobile device-assisted EMA has also been suggested to have the potential of reducing participant burden and recall bias [44].

Our review found daily diaries were combined with wrist-worn ActiGraph for assessing sleep outcomes. This is consistent with a previous review that established subjective sleep ratings are most generally measured using sleep diaries, and objective sleep parameters were measured using actigraphy [48]. This finding suggests the feasibility of using wearable devices in EMA studies among the rotation work population; ActiGraphs were worn during both working hours and off-shift periods throughout study periods.

Studies assessing sleep and sleepiness/fatigue combined both subjective and objective measurements, where in one study [25], subjective measures were used to confirm and complement missing objective assessments, and in another study [36], assessments were combined to determine sleep outcomes. Objective measurements could support removing the information bias associated with self-reported measurements [49]. However, evidence in the current review [26] and broader literature [50] have suggested subjective measurements of sleep and sleepiness may be correlated with objective measurements.

### 4.3 Protocol compliance and analytical methods

Compliance rates were generally inadequately reported; a compliance rate of 80.3–87% [11,38] was reported for paper and pencil diaries, consistent with the rate of 80% considered to be representative of the daily lives of participants [18]. Compliance with pen and paper diaries is reportedly high but limited by participants reporting high false compliance to scheduled assessments [18,47]. The participation or response rate based on the included analytical sample size was high in our review; suggesting that more of the rotation workers are able to complete the minimum number of assessments set by studies to be included in their analysis. Ensuring high compliance to study protocols is regarded as important in EMA studies [18]; strategies including participatory design techniques, prompting/signaling and the training of study participants, employing inconspicuous objective assessments using electronic devices, monitoring and feedback, and providing incentives have been stated to help enhance compliance among participants [14,18]. In our review, compliance strategies were inconsistent and inadequately reported. Compliance rates are required and essential for evaluating the quality of data collected and the validity of findings reported by a study [18,20]. Compliance rates are also important in informing and enhancing prospective EMA study designs [18,20]. We recommend subsequent EMA studies in rotation workers report response and compliance rates.

EMA datasets are large and complex, and analyzing such data could be challenging [18,51]. The use of common analytical methods including aggregation strategies, repeated measures analysis of variance, and multiple regressions have been indicated to be generally suboptimal and could lead to incorrect inferences as they assume the same number of assessments available per individual (equal variance), and ignore the hierarchical nature of EMA data and treat all the assessments as if they were independent [51]. Evidence from our review showed varied analytical methods used in EMA studies. Though analytical approaches employed in studies are directed by the hypothesis being tested [18], mixed or multilevel models have been indicated to have considerable advantages for analyzing EMA data [51], including having the ability to handle ‘correlated data and unequal variances’ [52].

### 4.4 Strengths and limitations

The key strength of this review is that it is the first to systematically review the literature and employed standardized guidelines for reporting EMA studies (such as Checklist for Reporting EMA Studies) to characterize the methodology of EMA studies assessing health outcomes among rotation workers in the resources and construction sector.

However, the limitations of this review need to be acknowledged. The review included only peer-reviewed publications and those in English, as such perhaps limited in scope and by publication bias. Some aspects of the included studies (e.g., compliance rates) were inadequately reported. Studies were mostly done among offshore oil and gas workers and in the offshore setting. This may limit the generalization of evidence on EMA methods and procedures to other onshore rotation work settings due to contextual working environment-specific differences. As such, more EMA studies among onshore rotation workers (e.g., mining and construction sectors) and settings are needed. Most of the studies assessed sleep and fatigue, which may employ EMA techniques that may not be generalizable to other study outcomes, as such more EMA studies assessing other outcomes such as mental health outcomes and lifestyle behaviors are needed. Studies examined diverse outcomes and reporting strategies, as such quantitative synthesis was limited.

## 5 Conclusion

The review revealed the common use of both daily diary and with-in day fixed interval contingent designs with continuous assessments, increasing the use of electronic EMA delivery techniques (website/online based diaries and wearable devices), and suggested data collection could be done over more than 1 monitoring periods and across the on-and off-shift phases of the rotation work roster with high participation/response rate.

Nonetheless, there were inconsistent or inadequate reports of prompting strategies and compliance-related information among the reviewed studies. This suggests the need for future EMA studies assessing the health outcomes of rotation workers to adequately report prompting strategies and compliance-related information. This will help in understanding the feasible prompting strategies and rotation workers' compliance to EMA protocols and help plan subsequent EMA study designs. More EMA studies particularly within day interval contingent designs are needed to further investigate psychological states and lifestyle behaviors, to clarify the achievability of EMA methods in assessing such outcomes among rotation workers in the resources industry. The most common assessment methods are one-off daily assessments due to the nature of rotation work; however, further studies are required to demonstrate the feasibility of methods such as event-based and random multiple prompts/assessments during working hours in the rotation work environment such as the mining environment.

## Funding

This study was supported by the Aberdeen-Curtin Alliance Curtin International Postgraduate Research Scholarship and Research Stipend Scholarship awarded to Bernard Yeboah-Asiamah Asare (Curtin ID: 17619778; Aberdeen ID: 51987326).

## Conflicts of interest

The authors declare no conflicts of interest.
