# Supplementary material for: Application of Ecological Momentary Assessment in Studies with Rotation Workers in the Resources and Related Construction Sectors: A Systematic Review
Source: Saf Health Work. 2022 Oct 15;14(1):10–6. doi: 10.1016/j.shaw.2022.10.004 (PMC10024174; doi:10.1016/j.shaw.2022.10.004)
Supplement: Multimedia component 1 [file mmc1.docx]

**Figure 1: Flow diagram of identifying and selecting studies for the systematic review** **(PRISMA 2020 flow diagram)**

**Identification of new studies via other methods**

**Identification of new studies via databases**

Records assessed for eligibility (*n*=6)

Records sought for retrieval (*n*=6)

Records identified from:

Citation searching (*n*=6)

Records of new included studies (*n*=4)

Total studies included in review (*n*=23)

Records not retrieved (*n*=1)

Records excluded (*n*=3739)

Records removed before screening:

Duplicates: (*n*=2332)

Records removed for missing of abstract: (*n*=261)

Records assessed for eligibility (*n*=80)

Records sought for retrieval (*n*=81)

Records screened (*n*=3820)

Records identified from: Databases (*n*=6972)

**Included**

**Screening**

**Identification**

Records excluded (*n*=2)

- Letter (*n*=1)
- Study method data overlap (*n*=1)

Records excluded (*n*=61)

- Not EMA design (*n*=29)
- Conference abstract/paper (*n*=8)
- Not industry of interest (*n*=8)
- Commuting arrangement not specified (*n*=5)
- Not in English Language (*n*=4)
- Letters (*n*=3)
- EMA design not clearly defined (*n*=2)
- Not outcome of interest (*n*=2)
