# Supplementary material for: Application of Ecological Momentary Assessment in Studies with Rotation Workers in the Resources and Related Construction Sectors: A Systematic Review
Source: Saf Health Work. 2022 Oct 15;14(1):10–6. doi: 10.1016/j.shaw.2022.10.004 (PMC10024174; doi:10.1016/j.shaw.2022.10.004)
Supplement: Multimedia component 2 [file mmc2.docx]

**Supplementary material 2**

**Table 1: Characteristics of EMA studies on health and related behavioural outcomes/predictors in rotation workers**

| **Author and Year** | **Setting and country** | **Sample size** | **Analytical sample** | **Age; mean(SD)** | **Study type** | **Outcomes** | **Predictors** |
| --- | --- | --- | --- | --- | --- | --- | --- |
| Albrecht and Anglim, 2018[21] | Construction  Australia | 79 | 52 | NR | Observational | Emotional exhaustion, engagement | Workload, emotional demand, social support, job autonomy |
| Bhuanantanondh et al., 2021[31] | Oil and gas  Thailand | 38 | 38 | 36.1(7.8) | Observational | Fatigue, sleep duration (hours) | Start and end of shift; day (day and night shift) |
| Bjorvatn et al., 1998[38] | Offshore oil and gas  Norway | 7 | 6 | 38.9 | Observational | Sleepiness; Sleep patterns | Day, work phase (on-shift and off-shift) |
| Bjorvatn et al., 1999[32] | Offshore oil and gas  Norway | 7 | 6 | 38.9 | Interventional | Sleepiness; Sleep patterns | Light, day, work phase (on-shift and off-shift) |
| Bjorvatn et al., 2007[33] | Offshore oil and gas; Norway | 38 | 17 | 42 | Interventional | Sleepiness; sleep patterns | Light, melatonin, day |
| Ferguson et al., 2010[34] | Mining  Australia | 42 | 29 | 37.4(6.8) | Observational | Sleep duration and fatigue | Shift type (day, night, day off) |
| Ferguson et al., 2011[10] | Mining  Australia | 111 | 35 | 40.3(10) | Observational | Fatigue, sleep | Fatigue: roster type, test time and sleep |
| Haward et al., 2009[35] | Offshore oil and gas  UK | 47 | 37 | 41.5(6.7) | Observational | Sleep problems (sleep quality, sleepy feeling, not enough sleep), tiredness, motion sickness symptoms (depression, anxiety, headache, dizziness, aches and pains, vomiting) | Motions (x-,y-,z-axes direction) of platform |
|  | | | | | | |  |

**Table 1: Characteristics of EMA studies on health and related behavioural outcomes/predictors in rotation workers (cont’d)**

| **Author and Year** | **Setting and country** | **Sample size** | **Analytical sample** | **Age; mean(SD)** | **Study type** | **Outcomes** | **Predictors** |
| --- | --- | --- | --- | --- | --- | --- | --- |
| Merkus et al., 2015[22] | Offshore oil and gas  Norway | 104 | 61 | 41.5(7.2) | Observational | Sleep quality, feeling rested, physical tiredness, mental tiredness, energy for activities | Shift type (day, night and swing shifts) |
| Merkus et al., 2015[37] | Offshore oil and gas  Norway | 52 | 29 | 43.5(9.8) | Observational | Smoking, Alcohol | Shift type (day and night shifts) |
| Merkus et al., 2017[36] | Offshore oil and gas  Norway | 104 | 61 | 41.5(7.2) | Observational | Leisure time physical activity | Shift type (day, night and swing shifts) |
| Muller et al., 2008[11] | Mining  Australia | 52 | 48 | 37 | Observational | Fatigue, sleep duration and quality, and alcohol intake | *Fatigue*: sleep duration, alcohol consumption, shift type (day 7 night shift) |
| Ots et al., 2021[12] | Offshore oil and gas  Netherlands | 50 | 36 | 44.3(11.1) | Observational | Physical activity, sleepiness | Roster phase (pre-, offshore and post-offshore phases). Sleepiness: PA, sleep quality |
| Paech et al., 2010[23] | Mining  Australia | 111 | 51 | 40.3(10) | Observational | Sleep (total sleep time and sleep quality) | Roster, shift type (day and night, off days)) |
| Rebar et al., 2018[6] | General FIFO  Australia | 64 | 64 | 40.39(10.34) | Observational | Sleep quality, exercise, nutrition quality, relaxation, alcohol intake, smoking, mental and physical impairments | Work phase (days on vs days off) |

**Table 1: Characteristics of EMA studies on health and related behavioural outcomes/predictors in rotation workers (cont’d)**

| **Author and Year** | **Setting and country** | **Sample size** | **Analytical sample** | **Age; mean(SD)** | **Study type** | **Outcomes** | **Predictors** |
| --- | --- | --- | --- | --- | --- | --- | --- |
| Riethmeister et al., 2018[24] | Offshore oil and gas  Netherlands | 60 | 42 | 42(12.1) | Observational | Sleep quality and sleepiness | Work phase (days on vs days off) |
| Riethmeister et al., 2018[25] | Offshore oil and gas  Netherlands | 60 | 42 | 42(12.1) | Observational | Fatigue (sleepiness), time in bed (sleep quality) | Time (pre-and post-shift) |
| Riethmeister et al., 2019[9] | Offshore oil and gas  Netherlands | 60 | 42 | 43.4(11.8) | Observational | Fatigue (Sleepiness), sleep duration and loss | Time (pre-and post-shift), days |
| Sadeghniiat-Haghighi et al., 2019[26] | Offshore oil and gas  Iran | 42 | 42 | 35.9(7.9) | Observational | Sleep duration and quality | Time (first week vs second week of a 2 week work period), shift type (fixed day, fixed night, swing: 7d/7n, standby |
| Saksvik et al., 2011[27] | Offshore oil and gas  Norway | 28 | 19 | 44.4(8.6) | Observational | Sleep (sleep duration, efficiency, and quality) | Shift type (day shift, night  shift, and swing shift), day |
| Thorne et al., 2008[29] | Offshore oil and gas  UK | 17 | 16 | 43.5(11.0) | Observational | Sleep duration and quality | Night shift start time  (18:00–06:00 h and 19:00–07:00 h) |
| Thorne et al., 2010[28] | Offshore oil and gas  UK | 14 | 10 | 47.5(9.0) | Interventional | Sleep duration and quality | Night shift start time  (18:00–06:00 h and 19:00–07:00 h) |
| Waage et al., 2012[30] | Offshore oil and gas  Norway | 28 | 15 | 44 | Observational | Sleepiness | Shift type (day shift, night  shift, and swing shift), day, time |

**Table 2: Methodological characteristics of the EMA studies on health and related behavioural outcomes in rotation workers**

| **Author and Year** | **EMA design/ approach** | **Method for EMAs delivery** | ***Monitoring periods; *Study duration/days** | ***Compliance rate; *Compliance enhancer (incentive)** | ***Assessment frequency (outcome); *prompts (frequency)** | ***Assessment period** | **Outcomes measures; Validity** | **Analysis method** |
| --- | --- | --- | --- | --- | --- | --- | --- | --- |
| Albrecht & Anglim, 2018[21] | Daily diary | Website/online diaries | 1; NR | NR; NR | Daily-Every 3 days; Yes (once) | On-shift days | Self-report; Multiple items; precedent | Multilevel models |
| Bhuanantanondh et al., 2021[31] | Daily diary  Interval contingent | Hand held device (tablet) | 1;14 days | NR;NR | Once daily (sleep duration), 2 times per day (fatigue); NR | On-shift days | Self-report and monitoring-Reaction response Time (RRT); precedent | Repeated measure ANOVA |
| Bjorvatn et al., 1998[38] | Daily diary  Interval contingent | Paper and pencil (Not specified) | 1; 21 days | NR; Yes (none) | Hourly (sleepiness), Daily (accumulated sleepiness and sleep) | On-and off-shift days | Sleepiness: self-report; single item. sleep pattern: multiple items; precedent | Repeated measure ANOVA |
| Bjorvatn et al., 1999[32] | Daily diary  Interval contingent | Paper and Pencil | 2; 42 days | NR; Yes (none) | Hourly (sleepiness); Daily (accumulated sleepiness and sleep); NR | On-and off-shift days | Sleepiness: self-report; single item. Sleep pattern: multiple items; precedent | Repeated measure ANOVA |
| ANOVA=Analysis of variance; CR=compliance rate; NR=not reported; PR=participation rate; ***Assessment period:** work roster cycle phase during which assessment was done  ***Assessment frequency**: number of times per day participants were assessed; ***Compliance enhancer:** measure undertaken to increase compliance to assessment schedules  ***Compliance rate**: the percentage of scheduled assessments to which participants completed; ***Method of EMA delivery**: method of administration of EMAs  * **Monitoring periods:** number of waves of data collection used in the study; ***Study duration:** the total number of assessment days each monitoring period lasted.  ***Participation or response rate:** the percentage of participants who completed the predetermined number of assessment; ***Prompts frequency**: number of times participants are alerted to answer assessment schedules | | | | | | | | |

**Table 2: Methodological characteristics of the EMA studies on health and related behavioural outcomes in rotation workers (cont’d)**

| **Author and Year** | **EMA design/ approach** | **Method for EMAs delivery** | ***Monitoring periods; *Study duration/days** | ***Compliance rate; *Compliance enhancer (incentive)** | ***Assessment frequency (outcome); *prompts (frequency)** | ***Assessment period** | **Outcomes measures; Validity** | **Analysis method** |
| --- | --- | --- | --- | --- | --- | --- | --- | --- |
| Bjorvatn et al., 2007[33] | Daily diary  Interval contingent | Wrist-worn device (Actigraph), handheld computer, paper and pencil | 1; 14 days | NR; Yes (none) | Hourly and daily (sleepiness)  Daily and  Continuous (sleep); NR | On-shift days | Sleepiness: Self-report and objective measure;  single item. Sleep pattern: multiple items; precedent | ANOVA |
| Ferguson et al., 2010[34] | Daily diary  Interval contingent diary | Wrist-worn device (Actiwatch) and Paper and pencil | 1;21-28 days | NR; NR | Daily and continuous (sleep); 2 times per day (fatigue); NR | On-and off-shift days | Sleep: Self-report and monitoring; multiple items.  Fatigue: self-report; single item; precedent | Mixed model ANOVA |
| Ferguson et al., 2011[10] | Daily diary  Interval contingent | Paper and pencil; Wrist-worn device (Actigraph); Hand held device (Palmpilot) | 1; 15-22 days | NR;NR | 2 time daily (fatigue)  Continuous (sleep) | On-and off-shift days | Reaction Response time (RRT) and monitoring | Linear mixed model |
| Haward et al., 2009[35] | Daily diary | Paper and pencil | 6; 84 days | PR:66-78%; NR | Daily; NR | On-shift days | Self-report; single-item; precedent | ANOVA and correlation |
| ANOVA=Analysis of variance; CR=compliance rate; NR=not reported; PR=participation rate. ***Assessment period:** work roster cycle phase during which assessment was done. ***Assessment frequency**: number of times per day participants were assessed. ***Compliance enhancer:** measure undertaken to increase compliance to assessment schedules. ***Compliance rate**: the percentage of scheduled assessments to which participants completed. ***Method of EMA delivery**: method of administration of EMAs. * **Monitoring periods:** number of waves of data collection used in the study. ***Study duration:** the total number of assessment days each monitoring period lasted. ***Participation or response rate:** the percentage of participants who completed the predetermined number of assessment; ***Prompts frequency**: number of times participants are alerted to answer assessment schedules | | | | | | | | |

**Table 2: Methodological characteristics of the EMA studies on health and related behavioural outcomes in rotation workers (cont’d)**

| **Author and Year** | **EMA design/ approach** | **Method for EMAs delivery** | ***Monitoring periods; *Study duration/days** | ***Compliance rate; *Compliance enhancer (incentive)** | ***Assessment frequency (outcome); *prompts (frequency)** | ***Assessment period** | **Outcomes measures; Validity** | **Analysis method** |
| --- | --- | --- | --- | --- | --- | --- | --- | --- |
| Merkus et al., 2015[22] | Daily diary | Paper and pencil | 1; 14 days | NR; NR | Daily; NR | On-shift days | Self-report; single-item; precedent | Generalized Estimating Equations |
| Merkus et al., 2015[37] | Daily diary | Paper and pencil | 1; 11days | NR;NR | Daily; NR | Off-shift days | Self-report; NR | Mann–Whitney U tests |
| Merkus et al., 2017[36] | Daily diary | Paper and pencil | 1;14 days | CR:80.3%; NR | Daily; NR | Off-shift days | Self-report; multiple items; precedent | Generalized Estimating Equations |
| Muller et al., 2008[11] | Daily diary  Interval contingent diary | Paper and pencil | 1;28 days | CR:87%, PR:95%; NR | Daily (sleep); 2 times per day (fatigue); NR | On-and off-shift days | Self-report; multiple items; precedent | t-tests and ANOVA; standardized parametric regression |
| Ots et al., 2021[12] | Interval contingent diary | Website/online; wrist-worn device (Actigraph) | 1;28 days | NR; NR | 2 times per day (sleepiness)  Continuous (physical activity, sleep quality) | On-and off-shift days | Self-report; single item (sleepiness); monitoring (PA, sleep quality) | Linear mixed models |
| ANOVA=Analysis of variance; CR=compliance rate; NR=not reported; PR=participation rate. ***Assessment period:** work roster cycle phase during which assessment was done. ***Assessment frequency**: number of times per day participants were assessed. ***Compliance enhancer:** measure undertaken to increase compliance to assessment schedules. ***Compliance rate**: the percentage of scheduled assessments to which participants completed. ***Method of EMA delivery**: method of administration of EMAs. * **Monitoring periods:** number of waves of data collection used in the study. ***Study duration:** the total number of assessment days each monitoring period lasted. ***Participation or response rate:** the percentage of participants who completed the predetermined number of assessment; ***Prompts frequency:** number of times participants are alerted to answer assessment schedules | | | | | | | | |

**Table 2: Methodological characteristics of the EMA studies on health and related behavioural outcomes in rotation workers (cont’d)**

| **Author and Year** | **EMA design/ approach** | **Method for EMAs delivery** | ***Monitoring periods; *Study duration/days** | ***Compliance rate; *Compliance enhancer (incentive)** | ***Assessment frequency (outcome); *prompts (frequency)** | ***Assessment period** | **Outcomes measures; Validity** | **Analysis method** |
| --- | --- | --- | --- | --- | --- | --- | --- | --- |
| Paech et al., 2010[23] | Daily diary | Wrist-worn device (Actigraphy) and Paper and pencil | 1;15-22 days | NR; NR | Daily; NR | On-and off-shift days | Self-report and monitoring; multiple items; precedent | Linear mixed models |
| Rebar et al., 2018[6] | Daily diary | Website/online diaries | 2; 14 days | NR; Yes (feedback) | Daily; NR | On-and off-shift days | Self-report; Single items; NR | Multilevel model |
| Riethmeister et al., 2018[24] | Interval contingent diary | Wrist-worn device (Actigraphy) and website/online diaries | 1; 28 days | NR; Yes (monitoring) | 2 times per day (sleep and sleepiness), continuous (sleep); NR | On-and off-shift days | Sleep: Self-report and objective measure; multiple-items.  Sleepiness: single item; precedent | Generalised linear and linear mixed models |
| Riethmeister et al., 2018[25] | Interval contingent diary | Wrist-worn device (Actigraph), Handheld device (iPad), and website/online diaries | 1; 14 days | NR; NR | 2 times per day (fatigue); continuous (sleep); NR | On-shift days | Fatigue/sleepiness: self-report and objective measure (RRT); Time in bed: single-item and objective measure; precedent | Generalised linear and linear mixed models |
| ANOVA=Analysis of variance; CR=compliance rate; NR=not reported; PR=participation rate. ***Assessment period:** work roster cycle phase during which assessment was done. ***Assessment frequency**: number of times per day participants were assessed. ***Compliance enhancer:** measure undertaken to increase compliance to assessment schedules. ***Compliance rate**: the percentage of scheduled assessments to which participants completed. ***Method of EMA delivery**: method of administration of EMAs. * **Monitoring periods:** number of waves of data collection used in the study. ***Study duration:** the total number of assessment days each monitoring period lasted. ***Participation or response rate:** the percentage of participants who completed the predetermined number of assessment; ***Prompts frequency**: number of times participants are alerted to answer assessment schedules | | | | | | | | |

**Table 2: Methodological characteristics of the EMA studies on health and related behavioural outcomes in rotation workers (cont’d)**

| **Author and Year** | **EMA design/ approach** | **Method for EMAs delivery** | ***Monitoring periods; *Study duration/days** | ***Compliance rate; *Compliance enhancer (incentive)** | ***Assessment frequency (outcome); *prompts (frequency)** | ***Assessment period** | **Outcomes measures; Validity** | **Analysis method** |
| --- | --- | --- | --- | --- | --- | --- | --- | --- |
| Riethmeister et al., 2019[9] | Interval contingent diary | Wrist-worn device (Actigraph) and website/online diaries | 1;14 days | NR; NR | 2 times per day (fatigue);  Continuous (sleep); NR | On-shift days | Fatigue/sleepiness: self-report and objective measure (RRT); single-item.  Sleep quality: objective measure; precedent | Linear mixed models |
| Sadeghniiat-Haghighi et al., 2019[26] | Daily diary | Wrist-worn device  Paper and pencil | 1; 14 days | NR; NR | Daily,  Continuous; NR | On-shift days | Self-report and objective measure; multiple items; NR | ANOVA |
| Saksvik et al., 2011[27] | Daily diary | Wrist-worn device (Actigraph) and Paper and pencil | 2; 56 days | PR:67.8%; NR | Daily,  Continuous; NR | On-and off-shift days | Self-report and objective measure; multiple items; precedent | ANOVA |
| Thorne et al., 2008[29] | Daily diary | Wrist-worn device (Actigraph) and Paper and pencil | 1; 7 days | NR; NR | Daily  Continuous; NR | On-shift days | Self-report and objective measure; single-item; NR | ANOVA |
| Thorne et al., 2010[28] | Daily diary | Wrist-worn device (Actigraph) and Paper and pencil | 1; 7 days | NR; NR | Daily  Continuous; NR | On-and off-shift days | Self-report and objective measure; single-item; NR | ANOVA |
| Waage et al., 2012[30] | Daily diary and Interval contingent diary | Hand-held computer and Paper and pencil | 2; 56 days | NR; Yes (monitoring and end day diary collection) | Hourly,  Daily; Yes (to start completing diaries) | On-and off-shift days | Self-report and objective measure; single-item; precedent | ANOVA |
| ANOVA=Analysis of variance; CR=compliance rate; NR=not reported; PR=participation rate. ***Assessment period:** work roster cycle phase during which assessment was done. ***Assessment frequency**: number of times per day participants were assessed. ***Compliance enhancer:** measure undertaken to increase compliance to assessment schedules. ***Compliance rate**: the percentage of scheduled assessments to which participants completed. ***Method of EMA delivery**: method of administration of EMAs. * **Monitoring periods:** number of waves of data collection used in the study. ***Study duration:** the total number of assessment days each monitoring period lasted. ***Participation or response rate:** the percentage of participants who completed the predetermined number of assessment; ***Prompts frequency**: number of times participants are alerted to answer assessment schedules | | | | | | | | |
